# Neural Correlates of Vocal Repertoire in Primates

**DOI:** 10.3389/fnins.2018.00534

**Published:** 2018-08-09

**Authors:** Jacob C. Dunn, Jeroen B. Smaers

**Affiliations:** ^1^Behavioural Ecology Research Group, Department of Biology, Anglia Ruskin University, Cambridge, United Kingdom; ^2^Biological Anthropology, Department of Archaeology, University of Cambridge, Cambridge, United Kingdom; ^3^Department of Anthropology, Stony Brook University, Stony Brook, NY, United States

**Keywords:** prefrontal cortex, cortical association areas, evolution of speech, vocal complexity, brain evolution, primate evolution, language, primates

## Abstract

Understanding the nature of the relationship between vocal complexity and brain architecture across non-human primates may help elucidate some of the key elements underlying the evolution of human speech. Here, we report a positive correlation between vocal repertoire size and the relative size of cortical association areas (governing voluntary control over behavioural output) in non-human primates. We further demonstrate that a hominid grade shift in the relative volume of cortical association areas coincides with a similar grade shift in the hypoglossal nucleus (which is associated with the cranial nerve that innervates the muscles of the tongue). Our results support a qualitative continuity in the neural correlates of vocal repertoire, but a quantitative discontinuity in the extent to which the neural system supporting speech is innervated by cortical association areas in great apes and humans.

## Introduction

Relative to humans, non-human primates (hereinafter “primates”) produce a very limited range of vocalisations. However, vocal repertoire varies widely among primate species, ranging from just two call types in Calabar angwantibos (*Arctocebus calabarensis*) to at least 38 call types in bonobos (*Pan paniscus*) (McComb and Semple, 2005). Understanding the evolutionary basis for such variation in vocal repertoire among our closest relatives may provide important insight into how a communication system as complex as human speech evolved.

The basic layout of the larynx and vocal tract is highly conservative and homologous in both form and function among virtually all terrestrial mammals, including humans (Fitch, 2000, 2006, 2010; Fitch and Zuberbuhler, 2013; Fitch et al., 2016). This strongly suggests that differences in vocal repertoire among primate species do not result from differences in vocal tract morphology (Fitch et al., 2016; Boë et al., 2017). Indeed, the macaque vocal tract has recently been shown to be “speech ready,” i.e., capable of producing an adequate range of speech sounds to support spoken language (Fitch et al., 2016). However, despite many attempts, no non-human primate has ever been trained to produce speech sounds.

One key factor that has been suggested to influence vocal repertoire is neural control (Fitch, 2010). This hypothesis posits that the reason primates are incapable of producing speech is because they lack adequate brain mechanisms to control and coordinate vocal production. The possibility has long been recognised; after examining the comparative data on vocal anatomy, Darwin concluded that “the development of the brain has no doubt been far more important” (Darwin, 1871). Nevertheless, this hypothesis has received surprisingly little attention, and, to date, there have been no comparative studies relating vocal repertoire to neuro-anatomy in primates.

Here, we test the hypothesis that vocal repertoire size in primates is associated with neural substrates of increased voluntary control over behavioural output. Two neural features are crucial in this regard: (1) the cortical association areas that govern voluntary control over behavioural output (Fuster, 1997; Miller, 2000); and (2) the brainstem nuclei that are involved in the neural control of the orofacial muscles (Sherwood et al., 2005). If voluntary control is an important element in vocal complexity and, by extension, the evolution of speech, we might expect these two neural features to be associated with each other and with vocal repertoire size. We also test for associations between vocal repertoire size and measures of both overall brain size and its main gross anatomical substructure, the neocortex.

## Materials and Methods

### Vocal Repertoire

We obtained data on primate vocal repertoire (the number of acoustically different calls that a species gives) from the literature. Following (McComb and Semple, 2005), we applied a series of rules when collecting data on vocal repertoire in order to make comparisons across species as systematic as possible. We only considered studies that reported the whole adult repertoire (excluding calls given exclusively by infants and juveniles) and which distinguished the calls on the basis of their acoustic structure. This led to the exclusion of studies that did not report the complete repertoire, or that only distinguished calls by the context in which they were given. To make all data consistent with that reported in (McComb and Semple, 2005), we did not consider lip smacking, teeth chattering/grinding or vomiting as part of the repertoire, and only included vocalisations that consisted of multiple units if any of these units had not previously been included as a distinct call in the repertoire. After applying these rules to each study, the vocal repertoire that we included in our analysis often differed from that reported in the original study. Humans were not included in these analyses, owing to the difficulty in quantifying vocal repertoire. Raw data are reported in Supplementary Table S1.

### Brain Region Volumes

We obtained brain region volumes (i.e., volumes of gross-anatomical structures and of areas within those structures) from published studies (Stephan et al., 1981; Sherwood et al., 2005; Smaers et al., 2010, 2011, 2013), all of which report data for the same specimens housed at the Vogt Institute for Brain Research (Zilles et al., 2011). We analysed three cortical association areas – the prefrontal cortex, the frontal motor cortex, and the temporal-parietal cortex; three brain stem nuclei – the trigeminal, facial, and hypoglossal nuclei; as well as brain volume and neocortical volume. All raw data are provided in Supplementary Table S1.

### Measures of Relative Size of Brain Regions

We only considered brain region volumes after controlling for overall size by using control variables that are functionally and neuroanatomically relevant (Dechmann and Safi, 2009; Passingham and Smaers, 2014). We controlled brain regions for size by obtaining residuals from a regression analysis. For the relative volume of the brain size, we used body size as the independent variable. For the relative volume of the neocortex, we used the rest of brain size as the independent variable. For the relative volume of brain stem nuclei, we used the volume of the rest of the brainstem as the independent variable. For the relative volume of cortical association areas, we used cortical primary sensory areas as the independent variable. Raw data are reported in Supplementary Table S1. We used different independent variables for these different neural regions considering the nature of their different functional and anatomical modularity and interconnectivity. It is crucial that the choice of appropriate comparative regions is made based on the functional and neuroanatomical realities of neural information processing. It has long been established that information processing in the brain occurs in a hierarchical manner. Stimuli are initially mapped onto primary sensory areas, and information subsequently projects to higher order association areas (Passingham et al., 2016). Throughout this process, perception, and interpretation of stimuli becomes increasingly complex (Desimone and Schein, 1987; Miller and Cohen, 2001). When aiming to assess the neural substrates of complex cognitive processing, an approach that adequately accounts for the functional and neuroanatomical underpinnings of neural information processing is to compare regions at the top of the hierarchy of information processing (heteromodal association areas) to those at the beginning of the hierarchy (primary sensory areas) (Passingham and Smaers, 2014). This approach effectively compares the amount of complex information processing relative to the amount of sensory input and is different to the more traditional procedure of comparing the size of a brain region to the size of the rest of the brain. This more traditional procedure is undesirable because it underestimates changes in neural systems (Passingham and Smaers, 2014) and erroneously assumes that neural information processing is isolated in particular regions, hereby ignoring the well-established hierarchical nature of neural information processing. Although it is advised to only consider relative volumes based on neurobiologically meaningful comparisons, we also checked results when calculating relative volumes compared to “rest of brain” in order facilitate comparisons with previous studies. These analyses provided similar results.

### Group Size

One factor that is thought to act as a selection pressure driving increases in vocal repertoire is sociality. The “social complexity hypothesis” for communication posits that animals with complex social systems require more complex communicative systems to regulate interactions and relations among group members (Freeberg et al., 2012). Indeed, there is now evidence in a diverse range of animal taxa, in different communicative modalities, that complexity in social groups is related to, and/or can drive complexity in signalling systems (Freeberg, 2006; delBarco-Trillo et al., 2012; Krams et al., 2012; Pollard and Blumstein, 2012; White et al., 2012). For example, McComb and Semple (2005) found a positive association between vocal repertoire and both group size and grooming rate (a measure of the strength of social bonding between individuals in a group) among non-human primates. Therefore, there is a need to control for the confounding effect of social group size on vocal repertoire when analysing the relationship between brain architecture and vocal repertoire.

In most primates, individuals form clearly defined social groups, which are relatively stable over time. However, some species form temporary parties or subgroups during foraging (e.g., *Macaca fascicularis*, *Pan troglodytes*). Therefore, we have distinguished between three types of social groups: (1) the foraging group (ForGroup), being the animals that forage together; (2) the population group (PopGroup), being the animals that share a common range or at least come together frequently, usually sleep together, and among which foraging units have highly overlapping ranges [data from Clutton-Brock and Harvey (1977) and Nunn and van Schaik (2002) in both cases]; (3) for the sake of comparison, we also analysed the group size data originally reported in McComb and Semple (2005) (MSgroup), which represent a midpoint of a range given in Rowe (1996). This may underestimate group size when solitary, perhaps dispersing individuals, are included as the lower group size in the primary reference. We use PopGroup as our principle measure of group size in the main text, as this is the most biologically meaningful measure, and report equivalent results for ForGroup and MSgroup in Supplementary Table S2. Raw data are reported in Supplementary Table S1.

### Statistical Methods

Comparative data points are not expected to be independent because of their shared phylogenetic history. To account for this data non-independence we use phylogenetic generalised least-squares procedures to analyse our data. We used phylogenetic analysis of covariance (pANCOVA) (Smaers and Rohlf, 2016; Smaers and Mongle, 2018) to assess the occurrence of grade shifts among primate clades in the relative volumes of brain regions and phylogenetic generalised least squares regression analysis [pGLS; Rohlf et al. (2001)] with a likelihood-fitted lambda parameter (Pagel, 1999) to test for correlations between vocal repertoire size and the relative size of brain regions. We calculated confidence intervals following Smaers and Rohlf (2016). The phylogeny (Supplementary Figure S1) was taken from the 10k Trees Project (Arnold et al., 2010). All data were log transformed prior to analysis.

Before deriving allometric residuals, we followed standard statistical procedures to test for significant differences in intercepts and slopes before interpreting allometry (Sokal and Rohlf, 2012). When subgroups indicate either a difference in slope, or a difference in intercept they should be considered as part of different allometry (Rao and Toutenburg, 1999). Therefore, if a multi-grade allometry was found to be a significantly better fit to the data than a single-grade allometry (Smaers and Rohlf, 2016), residuals were derived from the ancestral grade. For completeness, we also ran analyses using a single allometry. These analyses provided similar results.

## Results

**FIGURE 1:**
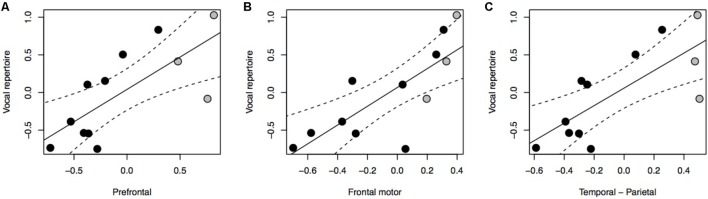
pGLS regressions of the residual volume of brain regions (**A**, prefrontal cortex; **B**, frontal motor cortex; **C**, temporal-parietal cortex) versus residual vocal repertoire. We represent 95% confidence intervals as dashed lines. Monkeys are represented in black, non-human apes in gray.

Our results indicate a significant positive association between vocal repertoire and the relative size of all three cortical association areas under investigation (**Figure 1A**: *F* = 11.23, *P* = 0.003, *df* = 2,10; **Figure 1B**: *F* = 15.05, *P* < 0.001, *df* = 2,10; **Figure 1C**: *F* = 12.72, *P* = 0.002, *df* = 2,10). Further analyses reveal no positive associations between vocal repertoire and the relative volume of the brain or neocortex, nor of any of the brain stem nuclei (Supplementary Figure S2). Using ForGroup, or MSGroup, instead of PopGroup as a control for vocal repertoire yielded equivalent results (Supplementary Table S2). Either considering or not considering the grade shift when calculating the relative size of brain region also produced equivalent results. Using absolute brain region volumes, rather than relative volumes, led to non-significant results (Supplementary Figure S3).

**FIGURE 2:**
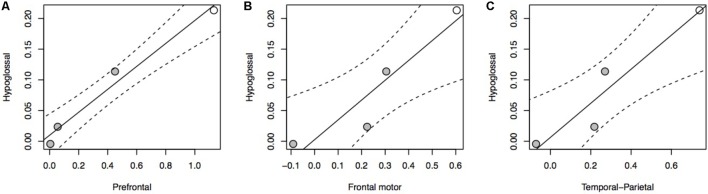
pGLS regressions of the residual volume of brain regions (**A**, prefrontal cortex; **B**, frontal motor cortex; **C**, temporal-parietal cortex) versus the residual volume of the hypoglossal nucleus in apes. We represent 95% confidence intervals as dashed lines. Non-human apes are represented in gray, humans in white.

The relationship between brain stem nuclei and cortical association areas was not significant when considering all species in the sample (Supplementary Figure S4). This primate-general trend, however, masks a significant relationship with prefrontal cortex and a marked positive trend with other cortical association areas in apes (**Figure 2A**: *F* = 81.85, *P* = 0.012, df = 2,2; **Figure 2B**: *F* = 14.5, *P* = 0.065, *df* = 2,2; **Figure 2C**: *F* = 17.39, *P* = 0.054, *df* = 2,2). This hominoid trend is confirmed by a significant difference in the hominid versus non-hominid allometry for both the cortical association areas (no difference in slope, but a difference in intercept; Smaers et al., 2017) and the relative size of the hypoglossal nucleus (difference in slope: *F* = 9.274, *P* = 0.004) (**Table 1**).

**Table 1:** Results from a phylogenetic ancova procedure on the relative size of cortical association areas and relative hypoglossal nucleus size, examining evidence for grade shifts among different primate clades.

pANCOVA			

Grouping	*df*	*F*	*P*
	**Prefrontal**		
	
Among groups (humans vs. great apes vs. others)	2,12	15.630	<0.001
Humans vs. others | great apes vs. others	1,12	30.622	<0.001
Humans vs. great apes | others	1,12	13.969	0.003
Great apes vs. others | humans	1,12	14.431	0.003

	**Frontal motor**		
	
Among groups (humans vs. great apes vs. others)	2,12	4.930	0.027
Humans vs. others | great apes vs. others	1,12	8.962	0.011
Humans vs. great apes | others	1,12	3.051	0.106
Great apes vs. others | humans	1,12	5.817	0.033

	**Temporal-parietal**		
	
Among groups (humans vs. great apes vs. others)	2,12	11.475	0.002
Humans vs. others | great apes vs. others	1,12	22.267	0.001
Humans vs. great apes | others	1,12	8.919	0.011
Great apes vs. others | humans	1,12	12.352	0.004

	**Hypoglossal**		
	
Among groups (humans vs. great apes vs. others)	2,42	3.866	0.029
Humans vs. others | great apes vs. others	1,42	7.355	0.010
Humans vs. great apes | others	1,42	4.427	0.041
Great apes vs. others | humans	1,42	2.071	0.158


## Discussion

The brain is central to the adaptive profile of any animal, as it underlies the capacity to modify behaviour. Therefore, natural selection shaping behavioural capacities is likely to be reflected in changes to the systems mediating those capacities. Indeed, there is strong evidence for specific brain adaptations for different locomotor strategies (de Winter and Oxnard, 2001), activity timing, diet, and habitat (Barton et al., 1995; Barton, 1996), spatial learning and memory (Jacobs et al., 1990), visual specialisation (Barton, 1998, 2004), sex differences in behaviour (Jacobs, 1996; Lindenfors et al., 2007), and variation in group size (Dunbar, 1992). Among birds, there is a significant correlation between the relative volume of song control nuclei and the number of song types typically found in the repertoire (Devoogd et al., 1993), however, to date there has been no similar research among mammals.

Several studies have highlighted changes in brain size as a key factor in species adaptation. For example, bigger relative brain size has been related to higher cognitive ability among primates (Deaner et al., 2007) and increased survival in mammals, birds, and reptiles (Sol et al., 2005, 2008; Amiel et al., 2011). Both relative brain size and relative neocortical volume have also been cited as important elements in the evolution of human speech, partly because of their positive correlation with group size (Aiello and Dunbar, 1993; Dunbar, 2010). Moreover, measures of overall brain size have recently been suggested to be associated to aspects of complex behaviour such as self-control (MacLean et al., 2014) and manipulation complexity (Heldstab et al., 2016). The use of overall brain size as a relevant measure to explain variation in behavioural complexity, however, remains contentious because it does not accurately represent critical neurobiological features such as modularity and interconnectivity (Chittka and Niven, 2009).

Here, we found no evidence that brain size or neocortex size (neither relative nor overall) were positively correlated with vocal repertoire size. However, when considering functionally specific, cytoarchitectonically defined areas of gross-anatomical structures, strong correlations were revealed. We focus on cortical association areas and demonstrate significant positive correlations with vocal repertoire among primates. Cortical association areas are areas within the neocortex that underlie the higher cognitive processing capacities often considered to be the foundation for the complex forms of behaviour observed in primates (Miller, 2000). The prefrontal cortex, for example, specifies the spatial and object goals of action, which can include a series of goals in the context of planning or performing sequences of action (Mushiake et al., 2006; Shima et al., 2007; Yamagata et al., 2012). Prefrontal-parietal function has been argued to aid in reducing errors during foraging choices and to provide a context for generating foraging goals (Genovesio et al., 2014). Overall, the nature of the processing capacities that underlie the cortical association areas can be understood as an increased voluntary control over behavioural output governed by an increased foresight, insight, and hindsight in the potential relevance of environmental cues (Fuster, 1997; Miller and Cohen, 2001; Passingham et al., 2016). Our findings that the relative sizes of cortical association areas are strongly correlated with vocal repertoire size suggest that vocal complexity coevolved with increases in such higher cognitive processing capacities. However, more research is necessary to understand the exact causal factors driving this relationship.

Particularly intriguing is the positive association between the hypoglossal nucleus and the cortical association areas in hominoids, most strongly observed with the prefrontal cortex. These results align with previous work reporting direct corticoefferent projections to the orofacial motor neurons in humans (Iwatsubo et al., 1990; Jürgens and Alipour, 2002; Simonyan and Jürgens, 2003), but an absence of such direct projections in macaques (Jürgens and Alipour, 2002). Direct neocortical projections to motor neurons in the human brain stem have traditionally been interpreted as indicative of increased cognitive control over the muscles that are innervated by those motor neurons (Kuypers, 1958; Pearce et al., 2003; Rödel et al., 2003). Our results confirm these earlier findings and suggest that the increased corticoefferent projections in hominoids and humans may stem primarily from cortical association areas (and the prefrontal cortex in particular). The positive trend in hominoids between cortical association areas and the hypoglossal nucleus suggests an increased cognitive control over the tongue in hominoids, with humans lying at the high end of this correlation.

One key difference that has been highlighted between human speech and primate vocalisations is that the former is learned while the latter is innate or reflexive (Fitch, 2000). In light of our results, this difference may be understood as an expansion of the voluntary control over vocalisations in hominoids and humans. Given that both the cortical association areas (Deacon, 1990) and the hypoglossal nucleus are significantly expanded in great apes and humans, it may be argued that the cortical association areas exerted more influence over the innervation of the tongue. Larger neural areas are known to exert more influence over overall brain function by means of increased connexional invasion (Deacon, 1990). This process allows hypertrophied areas to invade targets they did not innervate ancestrally, and/or to increase target innervation relative to the ancestral condition. Such new connexions may displace old connexions causing the hypertrophied areas to exert more influence over brain functioning.

Overall, our results support the idea of a qualitative continuity in the association between vocal repertoire and higher cognitive processing capacities by demonstrating a significant comparative correlation between cortical association areas and vocal repertoire size across non-human primates. Our results also suggest a quantitative discontinuity of prefrontal inputs supporting the transition from more reflexive vocalisations in non-human primates toward more learned complex vocalisations underlying human speech. We suggest that this quantitative discontinuity is based on increased innervation of cortical association areas to subcortical areas involved in speech production in great apes and humans (e.g., the hypoglossal nucleus). An interesting next step would be to evaluate whether such continuities/discontinuities are also found across other mammalian clades, with a particular focus on species capable of vocal learning. Recent research on the evolution of the cerebellum demonstrates that species capable of vocal learning exhibit significantly larger lateral cerebella than those that are not, suggestive of an evolutionary association between changes in cerebellar processing and cognition (Smaers et al., 2018).

Future work should continue attempting to match behavioural and brain data for a wide variety of species in a continued endeavour to investigate the evolutionary/comparative basis of behaviour. For example, cortical association areas may also be relevant in explaining other aspects of complex behaviour (e.g., deception, tool use, or self-control). Underlying this endeavour lies a continued effort to collect comparative neuroanatomical data. Data on the nucleus ambiguous (which is associated with the vagus nerve that innervates the larynx) would, for example, allow for a more direct test on the neural substrates of vocal production and the evolution of human speech (Fitch, 2011).

## Author Contributions

JD and JS participated in the design of the study, collected data, carried out analyses, and wrote the paper.

## Conflict of Interest Statement

The authors declare that the research was conducted in the absence of any commercial or financial relationships that could be construed as a potential conflict of interest.
