# Supplementary material for: Neural Correlates of Vocal Repertoire in Primates
Source: Front Neurosci. 2018 Aug 9;12:534. doi: 10.3389/fnins.2018.00534 (PMC6095195; doi:10.3389/fnins.2018.00534)
Supplement: Supplementary file 1 [file Data_Sheet_1.pdf]

Supplementary Material

Neural Correlates of Vocal Repertoire in Primates

Jacob C. Dunn* & Jeroen B. Smaers

*** Correspondence:** Corresponding Author: jacob.dunn@anglia.ac.uk

Supplementary Tables

Table S1. Raw data on vocal repertoire, group size, body size (g) and brain region volumes (mm^3) for all species

Species	VR	MS Group	Pop Group	For Group	Body -size	Brain	Neo-cortex	Pre-frontal	Frontal -- motor	Temporal - P arietal	Striate	Medulla	Trigeminal	Facial	Hypogloss
Alouatta-seniculus					6400	49009	31660	1014.8	1076.7	12394.0	1918.0	1593.3	2.3	1.7	1.6
Aotus-trivirgatus	6	3.5	3.3	3.3	830	16195	9950					675.3	0.9	1.2	0.9
Ateles-geoffroyi	24	19.75	22.7	3.9	8000	101034	70856	2772.5	3119.0	32933.0	3753.0	1833.7	1.8	1.7	1.8
Avahi-laniger					1285	10490	4813					553.4	0.9	1.1	0.7
Avahi-occidentalis					860	9670	4443					508.5	0.8	0.8	0.6
Callicebus-moloch	11	3.5	3.3	3.3	900	17944	11163					786.8	1.0	1.2	1.0
Callimico-goeldii	28	5	7.4	7.4	480	11000	6476					460.1	0.6	0.5	0.5
Callithrix-jacchus	13	8.9	8.2	8.2	280	7241	4371					318.2	0.5	0.5	0.4
Callithrix-pygmaea	16	6.4	7.9	7.9	140	4302	2535					185.0	0.3	0.3	0.3
Cebus-albifrons					3100	66939	46429	1433.9	1976.8	21024.0	3764.0	1738.3	2.2	2.5	2.0
Cercopithecus-ascanius	5	21	22	22	3400	63505	45166	1153.5	1351.3	16060.0	4272.0	1802.1	2.1	2.6	2.0
Cercopithecus-mitis	7	41	17.1	17.1	6300	70564	49933	1379.9	1852.4	19385.0	4232.0	1998.9	2.1	3.1	2.1
Cheirogaleus-major					450	6800	2938					381.6	0.5	0.8	0.9
Cheirogaleus-medius					177	2961	1221					202.4	0.3	0.5	0.6
Daubentonia-madagascariensis	9	1.5	1.5	1	2800	42611	22127					1517.1	1.9	2.0	2.2
Erythrocebus-patas					7800	103167	77141	1560.2	2552.1			2615.7	2.3	4.6	2.4
Eulemur-fulvus-fulvus	11	15.5	8.5	8.5	1400	22106	12207					909.4	1.0	1.4	1.5
Galago-senegalensis	18	2	3.5	1	186	4512	2139					254.3	0.3	0.6	0.4
Galagoides-demidoff	8	3.5	3.5	1	81	3203	1568					169.1	0.2	0.5	0.3
Gorilla-gorilla-	16	9	15.8	15.8	105000	470359	341444	11641.7	14854.1	125539.0	12062.0	8393.9	9.5	13.3	10.2
Homo-sapiens					65000	1251847	1006525	46322.9	29012.6	271891.0	14787.0	9699.8	7.7	13.0	14.4
Hylobates-lar					5700	97505	65800	2588.2	2744.4	28424.0	4574.0	2249.2	2.0	2.1	2.4
Indri-indri	9	4	4.3	4.3	6250	38300	20114					1342.0	1.7	2.5	1.8
Lagothrix-lagotricha	6	33	28	12.1	5200	95503	65873	1887.2	2433.6	24188.0	4856.0	2109.5	2.6	2.7	2.1
Lepilemur-ruficaudatus	6				915	7600	3282					449.3	0.7	0.9	1.2
Lophocebus-albigena	5	9.5	15.5	15.5	7900	97603	68733	2310.7	3925.9	29102.0	5420.0	2670.6	3.3	4.3	3.1
Loris-tardigradus	5	3	3	1	322	6269	3524					287.2	0.5	0.6	0.5
Macaca-mulatta					7800	87896	63482					2329.1	3.8	4.9	3.9
Microcebus-murinus	8	3	2.5	1	54	1680	739.51					99.0	0.1	0.3	0.3
Miopithecus-talapoin	12	45	92.6	92.6	1200	37776	26427	1018.1	906.2	12218.0	2339.0	1034.8	1.5	1.9	1.2
Nasalis-larvatus	4	9	10.8	10.8	14000	92797	62685	812.7	1749.0			2945.2	2.8	4.4	2.2
Nycticebus-coucang	8			1	800	11755	6192					528.1	1.0	1.0	0.8
Otolemur-crassicaudatus					850	9668	4723					539.9	0.9	1.4	0.8
Pan-paniscus	38	125	43.4	7.6				7955.8	8355.5						
Pan-troglodytes	29	26.5	47.6	5.6	46000	382103	291592	14209.4	13406.6	112750.0	10593.0	6027.4	5.1	12.3	7.8
Papio-anubis	6	50	69	69	25000	190957	140142	3920.8	4685.6	45240.0	10001.0	5171.2	6.2	7.4	4.6
Perodicticus-potto	5	1.5	1	1	1150	13212	6683					679.7	1.1	1.3	1.3
Piliocolobus-badius	17	49.5	30.2	30.2	7000	73818	50906	1591.3	2260.2	22629.0	3053.0	2007.1	2.2	2.3	1.5
Pithecia-pithecia	12	2.9	3.5	3.5	1500	32867	21028	600.4	819.1	9242.0	1679.0	1008.9	1.3	1.3	1.4
Pongo-pygmaeus	10	2	1.5	1.5				8595.7	6353.1	76924.0	7008.0				
Propithecus-verreauxi					3480	25194	13170					1222.6	1.7	2.0	1.6
Pygathrix-nemaeus	8	14.5	7.5	7.5	7500	77000	48763					2206.1	2.2	2.8	1.6
Saguinus-midas					340	10300	5883					428.5	0.5	0.6	0.6
Saguinus-oedipus	33	7.4	5.3	5.3	380	9537	5894					413.3	0.5	0.5	0.6
Saimiri-sciureus	21	32	45	45	660	22572	15541					721.5	0.8	0.8	0.8
Tarsius-syrichta	8		3	1	125	3393	1768					206.9	0.4	0.8	0.3
Varecia-variegata-	13	10.5	5.4	3.1	3000	29713	15293					1420.0	1.6	2.7	3.1

Table S2. Equivalent results from main text on the relationship between vocal repertoire and brain structure/area volume, using alternative data on group size

Group Variable	Brain structure/area	slope	slope_st.err	slope_min	slope_max	intercept	intercept_st.err	intercept_min	intercept_max	Rsquare	Pvalue	Lambda	df
PopGroup	Brain	0.20	0.50	-0.82	1.22	-0.01	0.26	-0.54	0.52	0.01	0.69	0.57	28
	Neocortex	-0.58	0.66	-2.02	0.85	0.00	0.59	-1.28	1.28	0.06	0.39	1.00	28
	Prefrontal	0.94	0.23	0.42	1.46	-0.05	0.12	-0.31	0.20	0.62	0.00	0.00	10
	Frontal motor	1.38	0.27	0.78	1.99	-0.02	0.10	-0.25	0.20	0.72	0.00	0.00	10
	Non-frontal	1.29	0.29	0.65	1.93	-0.03	0.11	-0.27	0.21	0.67	0.00	0.00	10
ForGroup	Brain	0.04	0.51	-1.02	1.10	0.00	0.21	-0.45	0.44	0.00	0.94	0.29	28
	Neocortex	2.27	3.48	-5.60	10.14	-0.37	0.29	-1.02	0.27	0.05	0.53	0.00	28
	Prefrontal	0.66	0.28	0.02	1.29	0.03	0.14	-0.28	0.34	0.35	0.04	0.00	10
	Frontal motor	0.96	0.37	0.13	1.79	0.05	0.14	-0.25	0.35	0.40	0.03	0.00	10
	Non-frontal	0.89	0.37	0.07	1.71	0.05	0.14	-0.26	0.35	0.37	0.04	0.00	10
MSGroup	Brain	0.02	0.48	-0.97	1.02	-0.01	0.31	-0.63	0.62	0.00	0.96	0.71	27
	Neocortex	-0.10	0.60	-1.42	1.21	-0.07	0.54	-1.25	1.10	0.00	0.87	1.00	27
	Prefrontal	0.86	0.26	0.29	1.43	0.04	0.13	-0.24	0.32	0.53	0.01	0.00	10
	Frontal motor	1.25	0.32	0.53	1.96	0.07	0.12	-0.19	0.33	0.60	0.00	0.00	10
	Non-frontal	1.16	0.33	0.44	1.89	0.06	0.12	-0.22	0.33	0.56	0.01	0.00	10

Supplementary Figures

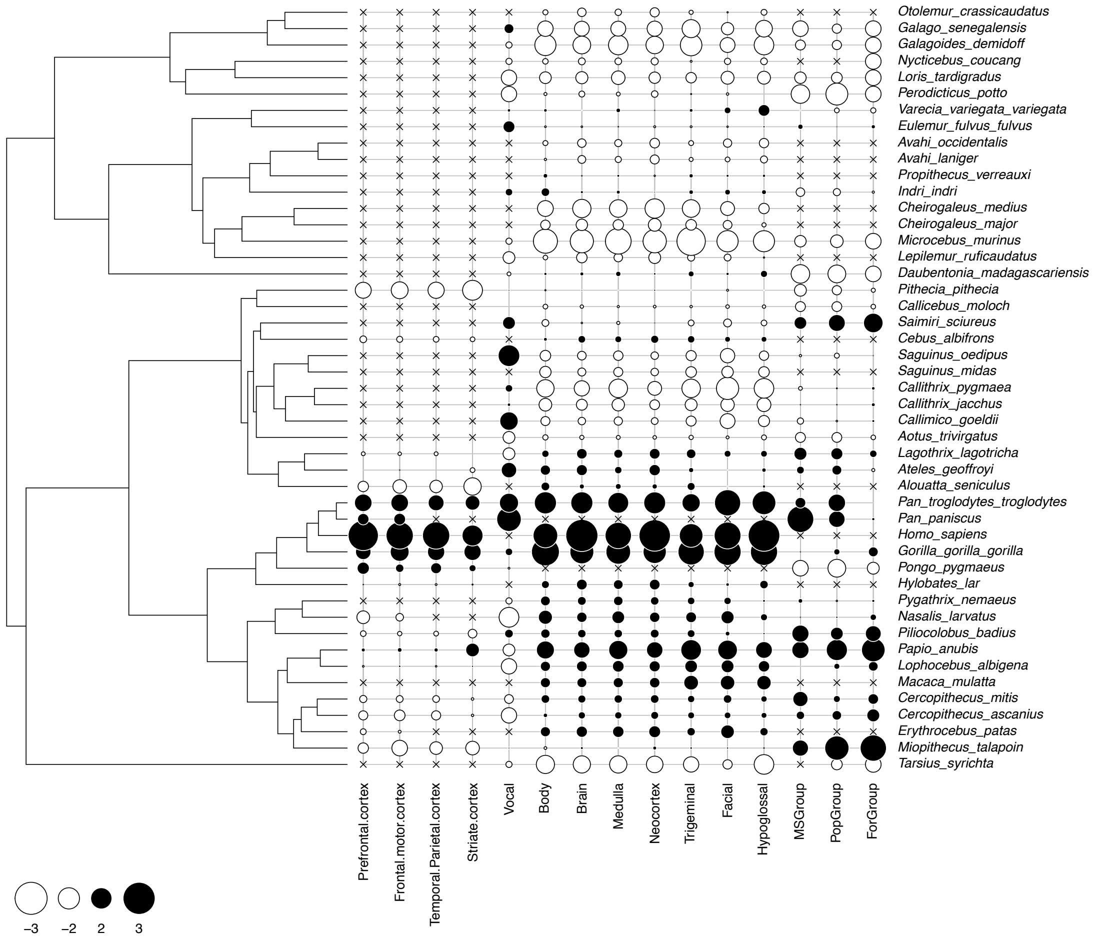

Figure S1. Phylogenetic tree of primate species studied and the relative magnitude of the traits studied. The size of the circle for each trait represents standardised scores of the raw data, black circles represent positive values and white circles represent negative values.

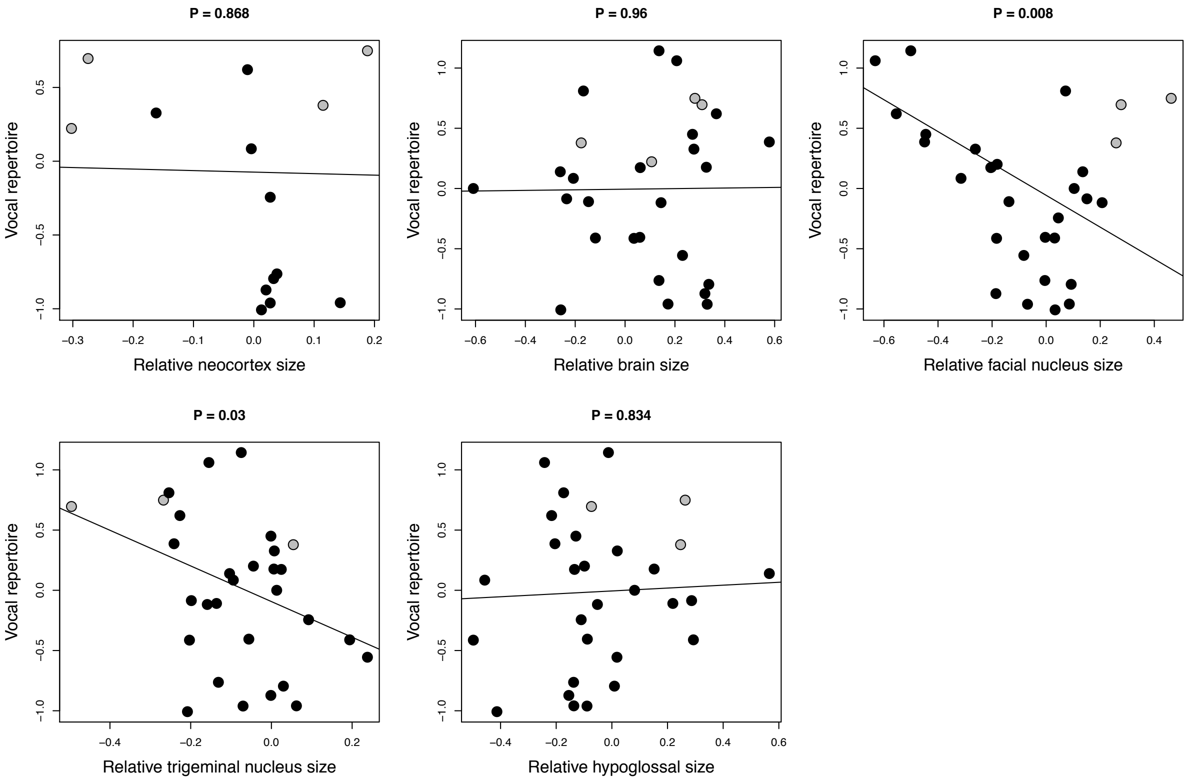

Figure S2. PGLS regressions of the residual volume of brain regions versus residual vocal repertoire. We represent 95% confidence intervals as dashed lines. Monkeys are represented in black, non-human apes in grey.

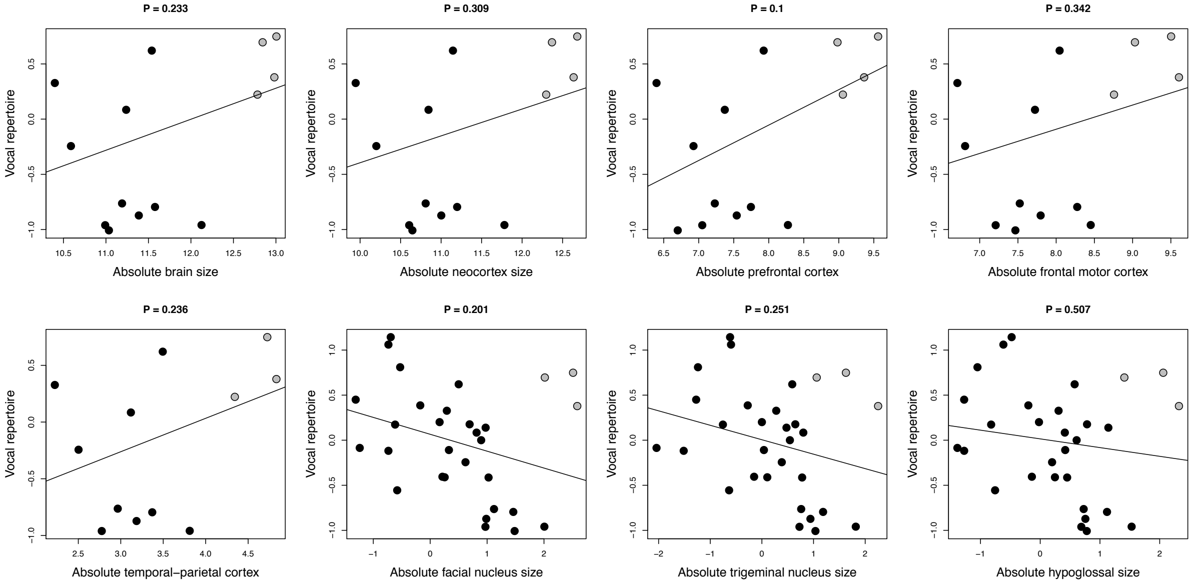

Figure S3. PGLS regression of absolute brain region volumes versus residual vocal repertoire. We represent 95% confidence intervals as dashed lines. Monkeys are represented in black, non-human apes in grey.

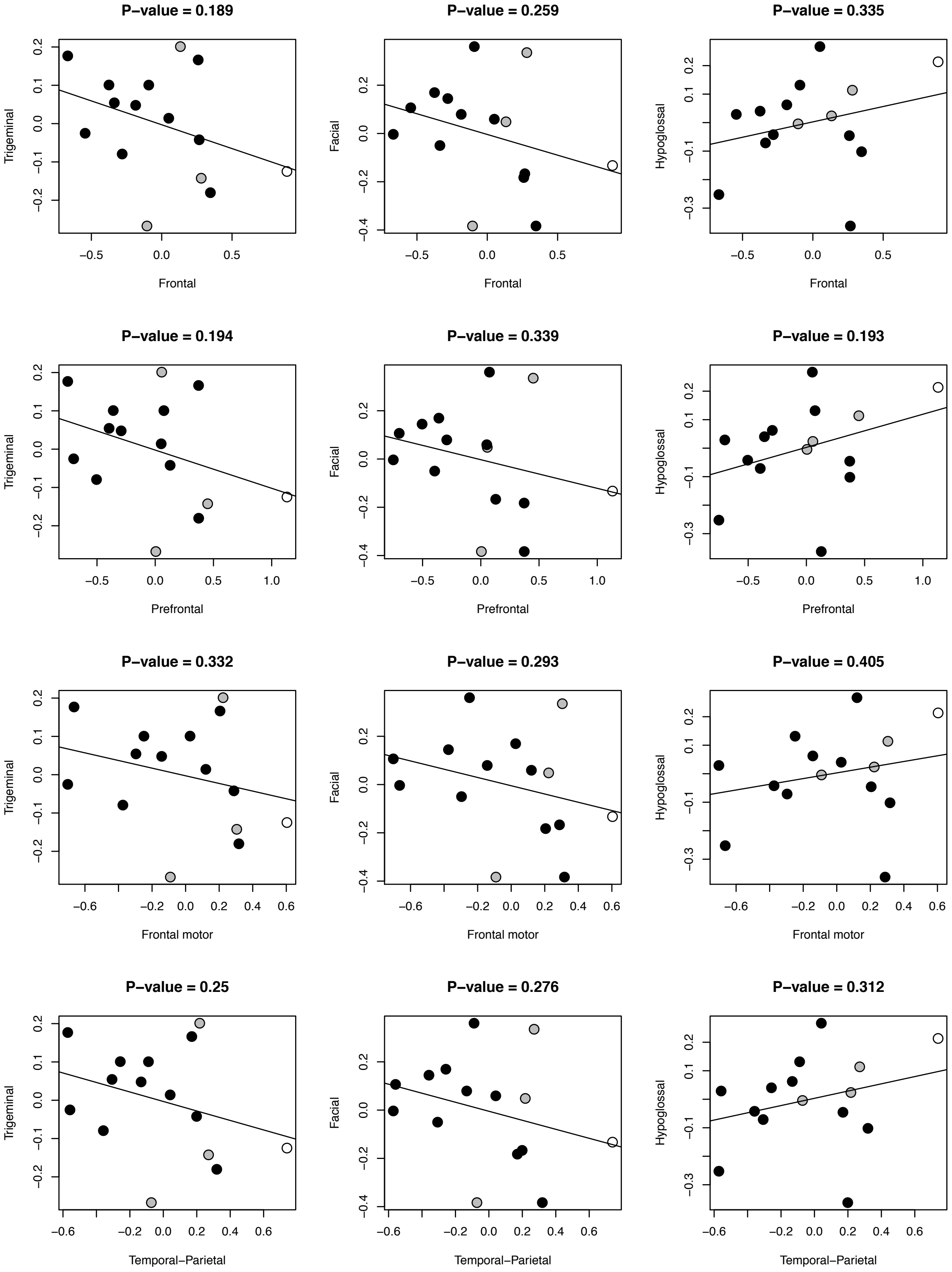

Figure S4. PGLS regression of the relative size of cortical association areas to the relative size of the brain stem nuclei. We represent 95% confidence intervals as dashed lines. Monkeys are represented in black, non-human apes in grey, humans in white.
